# Serological Response to the 2009 Pandemic Influenza A (H1N1) Virus for Disease Diagnosis and Estimating the Infection Rate in Thai Population

**DOI:** 10.1371/journal.pone.0016164

**Published:** 2011-01-25

**Authors:** Hatairat Lerdsamran, Chakrarat Pittayawonganon, Phisanu Pooruk, Anek Mungaomklang, Sopon Iamsirithaworn, Prasert Thongcharoen, Uraiwan Kositanont, Prasert Auewarakul, Kulkanya Chokephaibulkit, Sineenat Oota, Warin Pongkankham, Patummal Silaporn, Supaloek Komolsiri, Pirom Noisumdaeng, Tawee Chotpitayasunondh, Chariya Sangsajja, Witthawat Wiriyarat, Suda Louisirirotchanakul, Pilaipan Puthavathana

**Affiliations:** 1 Department of Microbiology, Faculty of Medicine Siriraj Hospital, Mahidol University, Bangkok, Thailand; 2 Bureau of Epidemiology, Department of Disease Control, Ministry of Public Health, Nonthaburi, Thailand; 3 Maharat Nakhonratchasima Hospital, Ministry of Public Health, Nakhonratchasima, Thailand; 4 Department of Pediatrics, Faculty of Medicine Siriraj Hospital, Mahidol University, Bangkok, Thailand; 5 The National Blood Center, The Thai Red Cross Society, Bangkok, Thailand; 6 Division of Preventive Medicine, Naval Medical Department, Royal Thai Navy, Chon Buri, Thailand; 7 Queen Sirikit National Institute of Child Health, Ministry of Public Health, Bangkok, Thailand; 8 Bamrasnaradura Infectious Disease Institute, Ministry of Public Health, Nonthaburi, Thailand; 9 Faculty of Veterinary Science, Mahidol University, Nakhon Pathom, Thailand; University of Georgia, United States of America

## Abstract

**Background:**

Individuals infected with the 2009 pandemic virus A(H1N1) developed serological response which can be measured by hemagglutination-inhibition (HI) and microneutralization (microNT) assays.

**Methodology/Principal Findings:**

MicroNT and HI assays for specific antibody to the 2009 pandemic virus were conducted in serum samples collected at the end of the first epidemic wave from various groups of Thai people: laboratory confirmed cases, blood donors and health care workers (HCW) in Bangkok and neighboring province, general population in the North and the South, as well as archival sera collected at pre- and post-vaccination from vaccinees who received influenza vaccine of the 2006 season. This study demonstrated that goose erythrocytes yielded comparable HI antibody titer as compared to turkey erythrocytes. In contrast to the standard protocol, our investigation found out the necessity to eliminate nonspecific inhibitor present in the test sera by receptor destroying enzyme (RDE) prior to performing microNT assay. The investigation in pre-pandemic serum samples showed that HI antibody was more specific to the 2009 pandemic virus than NT antibody. Based on data from pre-pandemic sera together with those from the laboratory confirmed cases, HI antibody titers ≥40 for adults and ≥20 for children could be used as the cut-off level to differentiate between the individuals with or without past infection by the 2009 pandemic virus.

**Conclusions/Significance:**

Based on the cut-off criteria, the infection rates of 7 and 12.8% were estimated in blood donors and HCW, respectively after the first wave of the 2009 influenza pandemic. Among general population, the infection rate of 58.6% was found in children *versus* 3.1% in adults.

## Introduction

There were 3 influenza pandemics occurring in the last century, i.e., Spanish influenza A (H1N1) in 1918, Asian influenza A (H2N2) in 1957 and Hong Kong influenza A (H3N2) in 1968 [1]. The influenza pandemic phase of this century, as declared by the World Health Organization (WHO) on 11^th^ June 2009, was caused by A (H1N1) virus [2], a reassortant derived from influenza viruses of 4 origins: classical swine, European swine, avian, and human influenza viruses [3]. Epidemiological studies of the 2009 pandemic showed that the disease is more common in children [4], [5]. Death mostly occurred in patients with underlying conditions, such as pregnancy, obesity, diabetes, hematological malignancy and cardiopulmonary dysfunction [6]–[9].

It is necessary to estimate and predict the magnitude of the pandemic in various regions worldwide, either by case based or serological based surveillance. However, the serological surveys were estimated to be approximately 10 times more sensitive than the clinical surveillance for determining infection rate of the pandemic virus [10], [11]. HI assay employing turkey erythrocytes was conducted; and the HI antibody titers ≥32 or ≥40 were established as the cut-off levels to estimate the infection rates in populations by various groups of investigators [10]–[13]. This cut-off titer was established based on the WHO guideline for vaccine evaluation which suggested HI antibody titers ≥40 as the levels indicating 50% protection [14], [15]. Moreover, microNT assay had been conducted in parallel in order to determine the protection correlation, and it was suggested that the HI antibody titer 40 was correlated to the NT titer 160 in adults or 40 in children [13].

On 10^th^ August 2010, WHO announced the beginning of the post-pandemic phase of the 2009 pandemic influenza. Nevertheless, epidemiological data from the Bureau of Epidemiology of Thailand suggested that only one fourth of the Thai population had been infected by this novel virus after it was introduced into Thailand at the beginning of May 2009 until December 2009. The data suggested that Southeast Asian countries and some other parts of the world might still be vulnerable to the new attack by that time.

The present study aimed to establish the cut-off HI and NT antibody titers that could differentiate between individuals with or without past infection by the 2009 pandemic influenza. We demonstrated that erythrocytes from goose yielded comparable HI antibody titers as those from turkey, an animal species that is not common in Southeast Asian countries. Moreover, we showed that it is necessary to treat human sera with receptor destroying enzyme (RDE) before running microNT assay. This RDE treatment is usually not included in the microNT protocol generally employed for testing human sera in most laboratories [10], [13], [16], [17]. Our established cut-off titers were applied in the seroepidemiological surveillance to estimate the infection rate in different groups of the Thai populations after subsidence of the first epidemic wave.

## Materials and Methods

### Ethical issues

This study was approved by two Ethical Committees: Siriraj Institutional Review Board, Faculty of Medicine Siriraj Hospital, Mahidol University and the Ministry of Public Health Review Board. Adult subjects signed in consent form for participation. With ascent from children subjects, their parents signed the consent form for them.

### Subjects

Serum samples tested in this study were collected from 5 groups of subjects. The first group comprised 80 patients with 2009 pandemic influenza as confirmed by real time reverse transcription- polymerase chain reaction employing the protocol of the US, Centers for Disease Controls [18]. Part of these patients were sent for disease diagnosis by the Bureau of Epidemiology, Department of Disease Control, Ministry of Public Health under public health emergency service; and part of them were sent anonymously from the clinic sites under the Southeast Asia Infectious Disease Clinical Research Network, Thailand. The second group comprised 100 anonymous blood donors of the National Blood Center, the Thai Red Cross Society, Bangkok. All were bled within the same day in September 2009. Small aliquots of blood were subjected to anti-HIV testing; and the leftovers were provided for this study. The third group comprised 258 healthcare workers (HCW) from two hospitals: Siriraj Hospital, Bangkok and Thammasat University Hospital in Pathum Thani, the neighboring province of Bangkok. These HCW were exposed to patients suspected of the 2009 pandemic influenza and/or to the laboratory confirmed cases during their duty. The fourth group comprised 222 general population from two provinces, Chiang Mai (696 km. north from Bangkok) and Nakhon Si Thammarat (780 km. south from Bangkok), whose ages were older than 5 years. These two provinces were selected based on highest numbers of reported cases in the region; and in each province, the random samples were collected in the community-based setting during the opinion survey in a district that high number of cases was reported. The test samples also included anonymously archival sera collected during pre-pandemic period from vaccinees whose ages were at range of 21–49 years. The vaccinees received inactivated influenza vaccine of the 2006 season which contained 15 µg of hemagglutinin antigen of A/New Caledonia/20/1999(H1N1)-like strain (A/New Caledonia), A/Wellington/1/2004(H3N2)-like strain, and B/Shanghai/361/2002-like strain (Government Pharmaceutical Organization-Merieux Biological Products Co., Ltd., Bangkok). Details regarding subjects who participated in this study are shown in Table 1.

**Table 1:** Subjects and time of specimen collection.

		Age (years)			
Subjects	No. of subjects	Mean	Median	Range	Time at specimen collection
**Vaccinees who received seasonal influenza**	71	33.6	31	21–49	Dec 2005 - Mar 2006 (pre-vaccination) Jan - Apr 2006 (post-vaccination)
**Patients**					
- Pediatrics	36	10.6	12.5	2–15	June 2009 – Feb 2010
- Adults	44	23.6	21	18–62	June 2009 – Feb 2010
**Blood donors**	100	35.7	34.5	17–60	Sep 2009
**Health care workers**	258	35.3	34	20–61	Oct 2009
**General population (Chiang Mai)**					
- Children	11	9.8	10	7–13	Dec 2009
- Adults	99	49.3	52	15–89	Dec 2009
**General population (Nakhon Si Thammarat)**					
- Children	18	10.1	10.5	5–14	Dec 2009
- Adults	94	49.5	48	15–87	Dec 2009

### Blood samples

Paired bloods were collected from the patients, and single blood samples were collected from the other groups of subjects. Acute blood samples were collected mostly within 7 days; meanwhile, the convalescent samples were collected at between 11 to 54 days after onset of illness. Serum was separated, aliquot and kept frozen at -20 C until tested.

Regarding archival sera, the pre-vaccinated blood samples were collected just before vaccination; and the post-vaccinated blood samples were collected at one month later.

### The study virus

A/Thailand/104/2009(H1N1) propagated in MDCK cells was used as the test virus for both HI and microNT assays. Full genomic sequence of this isolate can be retrieved from the GenBank database. The H genomic sequence of this virus was 99.7% identity to that of A/California/7/2009 pandemic virus (data not shown).

### Reference serum

A reference human serum from the National Institute for Biological Standards and Control (NIBSC), UK was used for standardizing our serological methods. Based on the investigation performed by various laboratories under the International Collaborative Study, this reference serum had the overall geometric mean titer (GMT) of 183 by HI and 516 by microNT assays (NIBSC package insert). The HI GMT titer 183 implies that the results of HI titers obtained from those laboratories varied between 160 and 320. Similarly, the NT GMT titer 516 implies that the results of NT titers varied between 320 and 640.

### Hemagglutination (HA) assay

HA assay was performed in order to measure the amount of hemagglutinin antigen present in the test virus suspension prior to running HI assay [17]. The test virus was serially twofold diluted with phosphate buffered saline (PBS) in a volume of 50 µl/well in duplicate. Fifty µl of 0.5% goose or 0.5% turkey erythrocyte suspension was added into the test wells and incubated for 30 minutes at 4 C before hemagglutinating result was determined. One HA unit of the test virus was defined as the highest virus dilution that displayed complete hemagglutinating activity.

### Hemagglutination inhibition (HI) assay

HI assay was performed as previously described [17], [19]–[21]. Fifty µl of the test serum were mixed with 150 µl of RDE (Denka Seiken, Tokyo, Japan) and incubated overnight in water bath at 37 C for eliminating the nonspecific inhibitors. This step was followed by heat inactivation at 56 C for 30 minutes, and removal of nonspecific agglutinator by absorbing with the test erythrocytes for 1 hour at 4 C. The replicating virus at final concentration of 4 HA units/25 µl was used as the test antigen; and goose or turkey erythrocytes were used as the indicator. The treated serum was twofold serially diluted in duplicate wells of a microtiter V shaped plate at an initial dilution of 1∶10; and 25 µl of the diluted serum were incubated with 25 µl of the test antigen for 30 minutes at room temperature. Thereafter, the reaction wells were added with 50 µl of 0.5% goose or 0.5% turkey erythrocyte suspension and further incubated for 30 minutes at 4 C before the HI antibody titers were determined. HI antibody titer is defined as the reciprocal of the highest serum dilution that completely inhibits hemagglutination reaction. Reference/positive control serum with known HI titer, the serum control and back titration of virus antigen were included in each run. For calculating GMT, the antibody titer <10 was assigned as 5, and the titer ≥2560 was assigned as 2560.

### Microneutralization (microNT) assay

ELISA based microNT assay was performed as described previously [17], [19]–[21]. The test sera were treated by any of the following two protocols. The first one was the standard protocol employing only heat inactivation of the native sera at 56 C for 30 minutes; and the second one employed RDE treatment similar to that mentioned above for HI assay. Briefly, 50 µl of the test serum were mixed with 150 µl of RDE and incubated overnight in water bath at 37 C followed by heat inactivation at 56 C for 30 minutes. The treated sera were twofold serially diluted in duplicate and incubated with the test virus at final concentration of 100TCID50/100 µl for 2 hours at 37 C. The serum-virus mixture was transferred onto MDCK monolayer maintained in minimum essential medium supplemented with trypsin TPCK (Sigma, St.Louis, MO.) for 24 hours. The reaction plate was tested by ELISA for presence of the viral nucleoprotein using mouse specific monoclonal antibody (Chemicon, Temecula, CA.) as the primary antibody and goat anti-mouse Igs (Southern Biotech, Birmingham, AL.) as the secondary antibody. Antibody titer is defined as reciprocal of the highest serum dilution that reduces ≥50% of the amount of viral nucleoprotein in the reaction wells as compared to the virus control wells. For calculating GMT, the antibody titer <10 was assigned as 5, and the titer ≥2560 was assigned as 2560.

## Results

### Goose and turkey erythrocytes yielded comparable HI antibody titers

**Figure 1:**
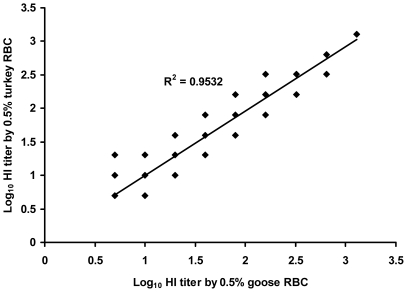
Correlation between HI antibody titers obtained from goose and turkey erythrocytes by HI assays.

In order to determine that goose and turkey erythrocytes yielded comparable HI antibody titers, the reference human serum from the NIBSC which contains HI antibody at GMT 183 was assayed in duplicate by 6 scientists using goose and turkey erythrocytes in parallel experiments. The HI antibody titer 160 was obtained from all 6 scientists as using either one of both erythrocyte species. The comparison was further extended to include the acute and convalescent serum samples from 53 patients as well as single serum samples from 100 HCW. The analysis on the total number of 206 serum samples showed that goose and turkey erythrocytes yielded comparable HI titers with *r* = 0.96 (Spearman's rank, p<0.0001) (Figure 1). The number of samples with HI antibody titers ≥40, as well as the ratio between convalescent to acute antibody titers, and the number of samples showing a fourfold or greater rise in HI antibody titer, were similar when goose or turkey erythrocytes were used (Table 2). Based on comparable HI titers obtained by the two erythrocyte species as well as our convenience to obtain goose erythrocytes; therefore, goose erythrocytes were employed in the subsequent experiments of our HI assay.

**Table 2:** Comparison between HI antibody titers obtained from goose and turkey erythrocytes.

Subjects	HI assay with	Number of sera test	Blood	GMT (95% CI)	No. with HI titers ≥40	No. with 4-fold rising Ab titer (%)	Ratio Conv./Acute
Patients	0.5% turkey RBC	Children = 29	Acute	14 (8–25)	7		
			Convalescent	111 (78–158)	28	23 (79.3)	7.6
		Adults = 24	Acute	15 (7–29)	6		
			Convalescent	87 (53–141)	22	19 (79.2)	5.8
	0.5% goose RBC	Children = 29	Acute	15 (8–27)	8		
			Convalescent	106 (72–157)	27	24 (82.8)	6.9
		Adults = 24	Acute	14 (7–28)	6		
			Convalescent	89 (55–145)	22	19 (79.2)	6.4
Health care workers	0.5% turkey RBC	Adults = 100	Single blood	9 (7–10)	14	NA	NA
	0.5% goose RBC	Adults = 100	Single blood	9 (7–11)	15	NA	NA

Note: A/Thailand/104/2009(H1N1) was used as the test virus. GMT  =  geometric mean titer, CI  =  confidence intervals, RBC  =  red blood cells,

NA  =  Not applicable

### RDE treated serum was required for microNT assay

We recognized that microNT assay using RDE untreated sera yielded an unusually high level of NT antibody to the 2009 pandemic virus in the test sera which had no HI antibody. Therefore, the serum samples were treated with RDE and retested again. The result showed that the RDE treated sera from all serum settings showed a marked decrease in level of NT antibody titer when compared to the RDE untreated samples (Wilcoxon Signed Ranks test, p<0.05) (Table 3). Therefore, the RDE treated sera were employed subsequently.

**Table 3:** Comparison between NT antibody titers obtained from RDE treated and RDE untreated sera.

				Number of cases at NT antibody of									
Subjects	No. of subjects	Blood samples	RDE treatment	<10	10	20	40	80	160	320	640	≥1280	GMT* (95% CI)
Patients	80	Acute	Yes	9	30	8	3	8	12	3	4	3	33 (23–47)
			No	0	0	0	7	18	21	22	5	7	191 (155–236)
	80	Convalescent	Yes	0	1	1	3	14	23	22	9	7	214 (172–267)
			No	0	0	0	1	2	11	36	22	8	380 (326–443)
General population	222	Single blood	Yes	15	87	46	33	24	10	5	2	0	21 (18–24)
			No	0	0	0	103	88	22	5	3	1	66 (61–72)
Vaccinees	71	Pre-vaccination	Yes	22	34	6	7	0	1	0	1	0	10 (8–13)
			No	0	0	1	5	33	27	2	3	0	110 (95–127)
	71	Post-vaccination	Yes	3	19	24	16	6	1	1	1	1	23 (18–28)
			No	0	0	0	0	24	34	8	4	1	152 (131–176)
Blood donors	100	Single blood	Yes	10	40	19	16	6	7	2	0	0	19 (16–24)
			No	0	0	0	1	29	60	10	0	0	138 (126–150)
Health care workers	258	Single blood	Yes	14	116	61	35	15	12	1	4	0	19 (16–21)
			No	0	0	0	10	81	127	34	5	1	138 (129–148)

*There are significant differences between GMT of NT antibodies in RDE treated and untreated sera from all 7 serum settings (Wilcoxon Signed Ranks test, p<0.05).

Moreover, the reference human serum from NIBSC which harbored NT antibody at GMT 516 was assayed in parallel by our two scientists using RDE treated serum as well as the untreated serum control in triplicate experiments. The GMT 640 was obtained either with the RDE treated or untreated serum.

### Cross-reactive antibody to the 2009 pandemic virus in pre-pandemic serum samples

Information about serological response in our vaccinees who received influenza vaccines during the pre-pandemic period had been published previously [22]. Among 71 tested sera, 98.6% developed a fourfold or greater rise in HI antibody titer against A/New Caledonia, a component of the immunizing vaccine (Table 4). Herein, those serum samples were investigated for serological response against the 2009 pandemic virus; and it was found that 16.9% of the vaccinees developed seroconversion as determined by HI assay. Six (8.4%) subjects seroconverted with HI titer rising from ≤10 to ≥40. Cross-reactive HI antibody titer 80 was found in one (1.4%) pre-vaccinated serum sample; nevertheless, post-vaccinated serum from this subject did not increase in antibody titer against the 2009 pandemic virus. At post-vaccination, number of subjects with the cross-reactive HI antibody ≥40 increased to 8 (11.3%). Regarding microNT assay, 16 (22.5%) developed a fourfold or greater rise in antibody titer (convalescent titer ≥40) in post-vaccination sera. Our study demonstrated broader cross-reactivity of NT antibody than HI antibody.

**Table 4:** Cross-reactive antibody to the 2009 pandemic A (H1N1) influenza virus in vaccinees who received trivalent influenza vaccine of the 2006 season (N = 71).

		Number of cases with antibody titer of										
Test viruses	Assays	<10	10	20	40	80	160	320	640	GMT (95% CI)	Post- to pre-vaccination ratio	No. with ≥4 folded rise in Ab titer a (%)
**A/New Caledonia/20/99-like (H1N1)**												
Pre-vaccination	HI	43	8	9	10	0	0	1	0	9 (7–11)		
Post-vaccination	HI	0	0	1	1	8	13	15	33	310 (254–379)	34	70 (98.6) b
**A/Thailand/104/09 (H1N1)**												
Pre-vaccination	HI	64	5	1	0	1	0	0	0	5 (5–6)		
Post-vaccination	HI	43	12	8	2	3	2	1	0	9 (7–11)	2	6 (8.4)
Pre-vaccination	microNT	22	34	6	7	0	1	0	1	10 (8–13)		
Post-vaccination	microNT	3	19	24	16	6	1	1	1	23 (18–28)	2	16 (22.5)

a Seroconversion with post-vaccination antibody titer ≥40 to the 2009 pandemic virus.

b A/New Caledonia/20/99 was used as the test antigen.

### HI and NT antibody response in patients infected with the 2009 pandemic influenza virus

**Figure 2:**
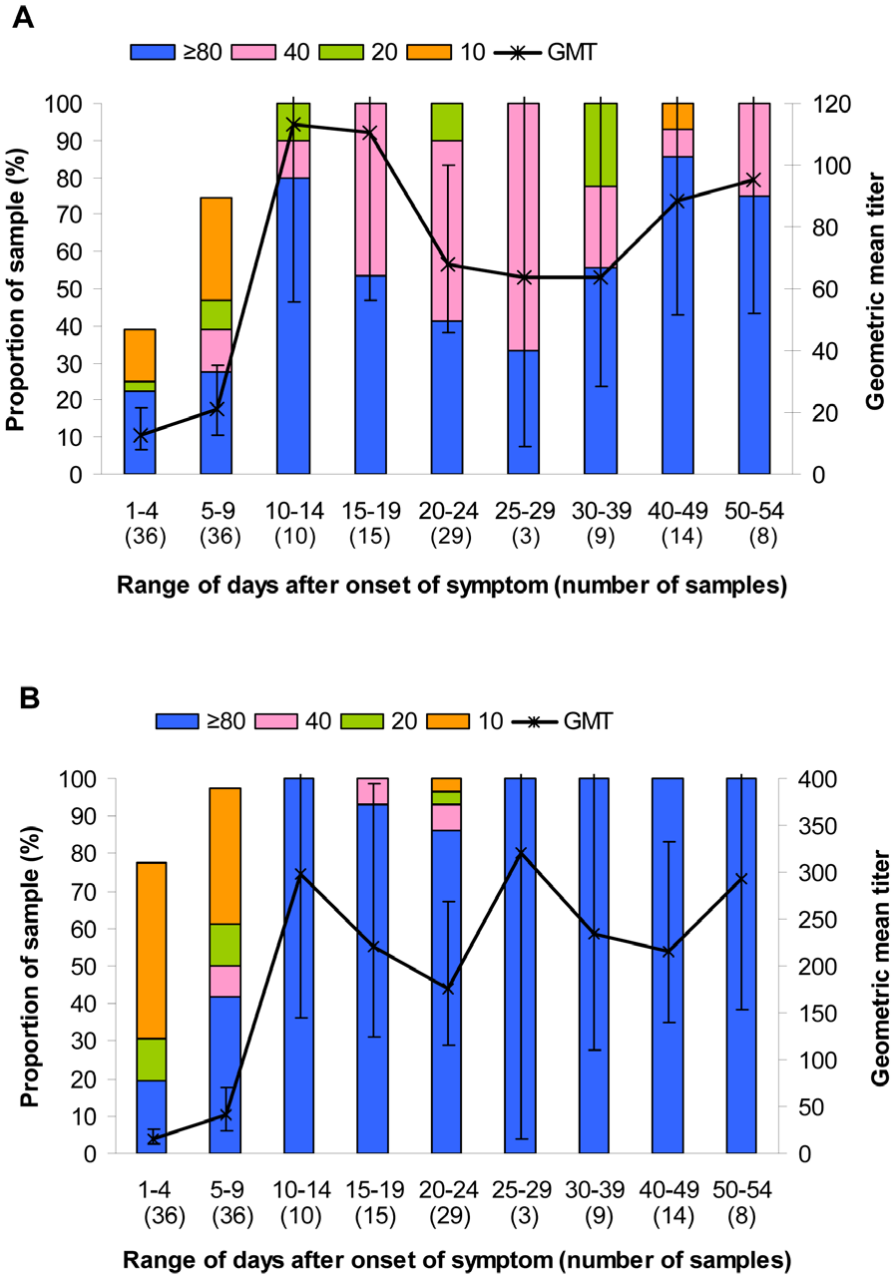
Antibody titers by date after onset of symptom. (A) HI antibody titer; (B) NT antibody titer. Colored stacked bars give the proportion with titers of 10, 20, 40 and ≥80 while the line denotes the geometric mean titer with error bars indicating 95% confidence intervals.

HI and NT antibody response in the patients by days after onset of illness is shown in Figure 2. Positive correlation with *r* = 0.85 (Spearman's rank, p<0.0001) was found between the two assays. Seroconversion or a fourfold or greater rise in HI or NT antibody titers could be demonstrated when convalescent blood samples were collected at 11 days earliest after disease onset. Among 36 pediatric cases, 32 (88.9%) developed seroconversion (Table 5). The other 4 cases already contained high HI titers ≥40 in their first blood samples. And among 32 seroconverters, 5 cases (13.9%) seroconverted with HI titers rising from <10 to 20; and the remaining 29 cases seroconverted with convalescent titers ≥40. Therefore, the HI titers ≥20 were found in 100%, and HI titer ≥40 were found in 86.1% of the pediatric patients. Similarly, the NT titers ≥20 were found in 97.2% and NT titer ≥40 were found in 94.4%.

**Table 5:** HI and NT antibody response in patients infected with the 2009 pandemic influenza virus.

			No. of cases at antibody titer of												
Patients	Assays	Blood samples	<10	10	20	40	80	160	320	640	1280	2560	GMT (95% CI)	Ratio of Conv./Acute	No. with ≥4 folded rise in Ab titers (%)
**Children (N = 36)**	HI	Acute	19	8	1	2	1	3	2	0	0	0	12 (7–19)		
		Conv.	0	0	5	12	10	3	1	4	1	0	78 (53–114)	6.3	32 (88.9%)
	microNT	Acute	5	20	2	1	5	3	0	0	0	0	16 (11–23)		
		Conv.	0	1	1	3	9	6	8	4	4	0	179 (119–269)	10.9	31 (86.1%)
**Adults (N = 44)**	HI	Acute	15	4	4	3	8	5	2	2	1	0	29 (17–48)		
		Conv.	0	1	0	16	15	6	3	2	1	0	83 (63–111)	38.1	24 (54.5%)
	microNT	Acute	4	10	6	2	3	9	3	4	2	1	58 (33–100)		
		Conv.	0	0	0	0	5	17	14	5	3	0	248 (199–310)	4.3	23 (52.3%)

Note: Number of pediatric patients with convalescent HI titers ≥20 = 36/36 (100.0%).

Number of adult patients with convalescent HI titers ≥40 = 43/44 (97.7%).

Among 44 adult patients, 43 (97.7%), developed HI titers ≥40 in their convalescent sera. There was one adult patient who could not develop significant HI antibody response (HI titer <10 and 10), although he possessed NT antibody titer of 20 and 80. The convalescent NT titers ≥80 were found in all adult patients.

### Estimation of the infection rate of the 2009 pandemic influenza after the first epidemic wave

Regarding titers of HI antibody found in the patients together with the data showing the absence of HI antibody in all except one pre-pandemic serum samples, the cut-off HI antibody titers ≥20 for pediatric cases and ≥40 for adult patients were established to indicate past infection by the 2009 pandemic virus. The established criteria had been used to estimate the infection rate of the 2009 pandemic influenza in various groups of populations; and it was found that 7.0% of blood donors and 12.8% of HCW had been infected with the pandemic virus by the end of the first epidemic wave (Table 6). Magnitude of the infection in the general population residing in the North and the South of Thailand was similar. In these two populations, the infection rates were much higher in children (17 of 29) than adults (6 of 193), i.e., 58.6 *versus* 3.1%, respectively.

**Table 6:** Estimation on the infection rates of the 2009 pandemic influenza in different groups of subjects after the first epide mic wave.

		No. of cases at antibody titer of									
Subjects	Assays	<10	10	20	40	80	160	320	640	Infection rate (%)	GMT (95% CI)
**Blood donors (N = 100)**	HI	81	7	5	7	0	0	0	0	7 (7.0)	6 (5–7)
	microNT	10	40	19	16	6	7	2	0		19 (16–24)
**Health care workers (N = 258)**	HI	177	26	22	16	12	5	0	0	33 (12.8)	8 (7–9)
	microNT	14	116	61	35	15	12	1	4		19 (16–21)
**General population (Chiang Mai, N = 110)**											
- Children (N = 11)	HI	2	0	1	4	2	2	0	0	9 (81.8)	37 (17–82)
- Adults (N = 99)	HI	86	8	2	3	0	0	0	0	3 (3.0)	5 (5–6)
- Children (N = 11)	microNT	0	1	1	0	2	2	3	2		141 (57–348)
- Adults (N = 99)	microNT	2	40	24	16	11	4	2	0		22 (18–26)
**General population (Nakhon Si Thammarat, N = 112)**											
- Children (N = 18)	HI	10	0	2	2	4	0	0	0	8 (44.4)	13 (7–24)
- Adults (N = 94)	HI	81	6	4	3	0	0	0	0	3 (3.2)	5 (5–6)
- Children (N = 18)	microNT	2	7	4	2	1	2	0	0		19 (11–32)
- Adults (N = 94)	microNT	11	39	17	15	10	2	0	0		17 (14–20)

Infection rate is determined by HI titer ≥40 in adults or ≥20 in children.

It was not easy to establish the cut-off NT antibody titer owing to presence of cross-reactive NT antibody at high titers in the pre-pandemic serum samples collected from vaccinees whose ages were under 50 years, and also in general people who had no HI antibody against the 2009 pandemic virus. Poorer correlation between HI and NT antibody was found in this group of subject (*r* = 0.32: Spearman's rank, p<0.0001) (data not shown).

## Discussion

Herein, HI and microNT assays that were suitable for a Southeast Asian country had been established to estimate the infection rate of the 2009 pandemic influenza in Thai people after subsidence of the first epidemic wave. HI assay has long been used for serodiagnosis of influenza virus infection, vaccine evaluation, and vaccine strain selection [16], [23], [24]. It was noted that sensitivity of the HI assay could be affected by the erythrocyte species employed. Stephensen, et al [25] previously reported that horse erythrocytes were more sensitive than turkey erythrocytes in the detection of HI antibody to H5N1 highly pathogenic avian influenza (HPAI) virus. Since there was difficulty in accessing both horse and turkey erythrocytes in Thailand, we previously looked for the alternative erythrocyte species and found that goose erythrocytes yielded comparable results in both HA and HI assays [19]. Similarly, the present study showed that goose erythrocytes could replace turkey erythrocytes for detection of HI antibody to the 2009 pandemic influenza virus. Our preliminary study on 206 serum samples as well as the reference serum from NIBSC demonstrated good correlation between HI titers employing either goose or turkey erythrocytes. This finding is an advantage for laboratories in Southeast Asian countries where goose erythrocytes have long been used in HI assay for diagnosis of dengue, chikungunya and Japanese encephalitis, the endemic diseases in this region. It was also demonstrated that the hemagglutination pattern of goose was also clearer than that of turkey erythrocytes.

A number of laboratories performed microNT in adjunct with HI assay in a seroepidemiological study, but the step of serum treatment with RDE was not included [10], [13]. In contrast, our study showed that NT GMT titer in the test sera without RDE treatment was significantly higher than that employed the RDE treated sera. Collectively, RDE is used for removal of nonspecific inhibitor from the test sera, in which its presence may lead to false positive result in HI as well as microNT assays as shown by this study. On the other hand, the presence of this nonspecific inhibitor did not affect our result on using RDE untreated sera in microNT assay for antibody against H5N1 HPAI virus. Cross-reactive H5N1 antibody, even at low level, was rare [20], [21], [26]. Therefore, it is postulated that HPAI H5N1 and the 2009 pandemic viruses bind to different species of nonspecific inhibitor.

Investigation in pre-pandemic serum samples demonstrated that NT antibody is broader in activity than HI antibody; however, both cross-reactive HI and NT titers could be increased by seasonal influenza vaccination. Similarly, Hancock, et al. [13] previously reported a fourfold or greater increase in cross-reactive antibody to the 2009 pandemic virus in archival serum samples from adult recipients of trivalent inactivated influenza vaccines during 2007–2009 seasons. A fourfold or greater increase in cross-reactive HI antibody was found in 7% among vaccinees of age 18–64 years, but it was as high as 22% for cross-reactive NT antibody. This cross- reactive antibody was rarely found in young children in their study. Our group previously reported that 5.2% of the elderly who received seasonal influenza vaccine seroconverted to HPAI H5N1 virus as determined by microNT assay [27]. Nevertheless, the studies from U.S. and Australia concluded that vaccination with seasonal influenza vaccine did not protect against the current pandemic [13], [28]. In contrast, partial protection conferred through seasonal influenza vaccination was reported by the other group of investigators [29]. Frequency of cross-reactive NT antibody was high in Thai and U.S. population [13]; and it was as low as 0.3% in Chinese [12]. However, it is well accepted that NT antibody activity is broader than HI antibody. HI antibody recognizes small epitopes in erythrocyte binding site, while NT antibodies recognize the epitopes in HA1 variable domain of the hemagglutinin molecule as well as the epitopes in HA2 domain which is conserved across influenza A subtypes [30]–[32].

Only one (2.3%) of our 44 adult patients failed to mount HI antibody titers ≥40 in their convalescent blood. Meanwhile, Miller, et al [10] demonstrated that HI assay failed to diagnose 10.9% of laboratory confirmed cases in their setting as the cut-off point ≥32 was employed. According to Millers, et al, the adult and pediatric patients were not separately analyzed; and the HI titers 32 or greater were found in 89.1% of their patients. In our study, if the data from pediatric and adult patients was pooled and analyzed together, the HI titer 40 or greater will be found in 92.5% of our patients. Regarding the study by Chen, et al [33], the HI titers 40 or greater were found in 93% of their adult patients if the convalescent blood samples were collected at peak between 25 and 29 days after onset of symptom.

Our study decided to use the HI antibody at cut-off titers ≥40 for adults and ≥20 for children to differentiate between individuals with and without past infection by the 2009 pandemic influenza. The cut-off titer for NT antibody could not be established because frequency of the cross-reactive NT titers was high as shown from the result of investigation in pre-pandemic sera obtained from vaccinees of age younger than 50 years, and additionally, from the high number of general adult population who had no HI antibody, but possessed NT titer ≥40. Based on our criteria, magnitudes of the 2009 pandemic influenza after the first epidemic wave were around 7% in blood donors and 12.8% in HCW. Eventually, the infection rate in general population was much higher in children than adults, i.e., 58.6% (17/29) *versus* 3.1% (6/193), which is suggestive of susceptibility of children and partial protection from pre-existing immunity in Thai adults. In United Kingdom, seroincidence rate of the 2009 pandemic influenza was also high in children [10]. Seroepidemiological data from Pittsburgh, U.S. showed that approximately 21% of population was infected following the second epidemic wave [34]. Estimation on the infection rates based on serological data may be affected by confounding factor of cross-reactive antibody-rising from seasonal influenza vaccination; and probably from pre-existing antibody against the 1957 influenza A (H1N1) virus. Nevertheless, this effect may be not drastic in the Thai population owing to less than 1% coverage of seasonal influenza vaccination among the Thai population. In addition, most of our adult subjects were younger than 50 years, therefore, cross-reactive antibody owing to previous infection by the 1918 influenza A (H1N1) virus was excluded.

Thailand reported the first two imported cases from Mexico in the beginning of May 2009. Subsequently, the virus was re-introduced into the country both by groups of tourists and Thai students who returned from Europe and America. The first epidemic wave began in late May, peaked in July and almost disappeared in November 2009. The first wave was followed by a short period of the second epidemic wave during December 2009 to April 2010 with peak in February. The third epidemic wave which lasted between June and October with peak in August 2010 was more serious than the second one. Serosurveillance nationwide will help the estimation for number of vulnerable people and immunity of the population to this pandemic virus. The 2009 pandemic monovalent vaccine was introduced into Thailand in December 2009; and trivalent vaccine containing the 2009 pandemic virus as a component has been introduced into the country in June 2010. Nevertheless, the vaccine coverage was less than 3% of the Thai population. Therefore, cross-reactive HI antibody due to the pandemic virus might have least effect on the estimated infection rate in the present study.

## References

[1] Morens DM, Fauci AS (2007). The 1918 influenza pandemic: insights for the 21st century.. J Infect Dis.

[2] Chan M (2009). http://www.who.int/mediacentre/news/statements/2009/h1n1_pandemic_phase6_20090611/en/index.html.

[3] Neumann G, Noda T, Kawaoka Y (2009). Emergence and pandemic potential of swine-origin H1N1 influenza virus.. Nature.

[4] Fraser C, Donnelly CA, Cauchemez S, Hanage WP, Van Kerkhove MD (2009). Pandemic potential of a strain of influenza A (H1N1): early findings.. Science.

[5] Novel Swine-Origin Influenza A (H1N1) Virus Investigation Team (2009). Emergence of a novel swine-origin influenza A (H1N1) virus in humans.. N Engl J Med.

[6] Hajjar LA, Mauad T, Galas FR, Kumar A, da Silva LF (2010). Severe novel influenza A (H1N1) infection in cancer patients.. Ann Oncol.

[7] Jamieson DJ, Honein MA, Rasmussen SA, Williams JL, Swerdlow DL (2009). H1N1 2009 influenza virus infection during pregnancy in the USA.. Lancet.

[8] Miller M, Viboud C, Simonsen L, Olson DR, Russell C (2009). Mortality and morbidity burden associated with A/H1N1pdm influenza virus: Who is likely to be infected, experience clinical symptoms, or die from the H1N1pdm 2009 pandemic virus? Version 2.. PLoS Curr.

[9] World Health Organization (2009). http://www.who.int/csr/disease/swineflu/assess/disease_swineflu_assess_20090511/.

[10] Miller E, Hoschler K, Hardelid P, Stanford E, Andrews N (2010). Incidence of 2009 pandemic influenza A H1N1 infection in England: a cross-sectional serological study.. Lancet.

[11] Reed C, Katz JM (2010). Serological surveys for 2009 pandemic influenza A H1N1.. Lancet.

[12] Chen H, Wang Y, Liu W, Zhang J, Dong B (2009). Serologic survey of pandemic (H1N1) 2009 virus, Guangxi Province.. China Emerg Infect Dis.

[13] Hancock K, Veguilla V, Lu X, Zhong W, Butler EN (2009). Cross-reactive antibody responses to the 2009 pandemic H1N1 influenza virus.. N Engl J Med.

[14] de Jong JC, Palache AM, Beyer WE, Rimmelzwaan GF, Boon AC (2003). Haemagglutination-inhibiting antibody to influenza virus.. Dev Biol (Basel).

[15] Eichelberger M, Golding H, Hess M, Weir J, Subbarao K (2008). FDA/NIH/WHO public workshop on immune correlates of protection against influenza A viruses in support of pandemic vaccine development, Bethesda, Maryland, US, December 10–11, 2007.. Vaccine.

[16] Rowe T, Abernathy RA, Hu-Primmer J, Thompson WW, Lu X (1999). Detection of antibody to avian influenza A (H5N1) virus in human serum by using a combination of serologic assays.. J Clin Microbiol.

[17] World Health Organization (2002). http://www.who.int/csr/resources/publications/influenza/en/whocdscsrncs20025rev.pdf.

[18] World Health Organization (2009). http://www.who.int/csr/resources/publications/swineflu/CDCRealtimeRTPCR_SwineH1Assay-2009_20090430.pdf.

[19] Louisirirotchanakul S, Lerdsamran H, Wiriyarat W, Sangsiriwut K, Chaichoune K (2007). Erythrocyte binding preference of avian influenza H5N1 viruses.. J Clin Microbiol.

[20] Dejpichai R, Laosiritaworn Y, Phuthavathana P, Uyeki TM, O'Reilly M (2009). Seroprevalence of antibodies to avian influenza virus A (H5N1) among residents of villages with human cases, Thailand, 2005.. Emerg Infect Dis.

[21] Kitphati R, Pooruk P, Lerdsamran H, Poosuwan S, Louisirirotchanakul S (2009). Kinetics and longevity of antibody response to influenza A H5N1 virus infection in humans.. Clin Vaccine Immunol.

[22] Auewarakul P, Kositanont U, Sornsathapornkul P, Tothong P, Kanyok R (2007). Antibody responses after dose-sparing intradermal influenza vaccination.. Vaccine.

[23] Ndifon W, Dushoff J, Levin SA (2009). On the use of hemagglutination-inhibition for influenza surveillance: surveillance data are predictive of influenza vaccine effectiveness.. Vaccine.

[24] Russell CA, Jones TC, Barr IG, Cox NJ, Garten RJ (2008). Influenza vaccine strain selection and recent studies on the global migration of seasonal influenza viruses.. Vaccine.

[25] Stephenson I, Wood JM, Nicholson KG, Zambon MC (2003). Sialic acid receptor specificity on erythrocytes affects detection of antibody to avian influenza haemagglutinin.. J Med Virol.

[26] Hinjoy S, Puthavathana P, Laosiritaworn Y, Limpakarnjanarat K, Pooruk P (2008). Low frequency of infection with avian influenza virus (H5N1) among poultry farmers, Thailand, 2004.. Emerg Infect Dis.

[27] Kositanont U, Wongsurakiat P, Pooruk P, Maranetra N, Puthavathana P (2010). Induction of cross-neutralizing antibody against H5N1 virus after vaccination with seasonal influenza vaccine in COPD patients.. Viral Immunol.

[28] Kelly H, Grant K (2009). Interim analysis of pandemic influenza (H1N1) 2009 in Australia: surveillance trends, age of infection and effectiveness of seasonal vaccination.. Euro Surveill.

[29] Garcia-Garcia L, Valdespino-Gómez JL, Lazcano-Ponce E, Jimenez-Corona A, Higuera-Iglesias A (2009). Partial protection of seasonal trivalent inactivated vaccine against novel pandemic influenza A/H1N1 2009: case-control study in Mexico City.. BMJ.

[30] Gocník M, Fislová T, Sládková T, Mucha V, Kostolanský F (2007). Antibodies specific to the HA2 glycopolypeptide of influenza A virus haemagglutinin with fusion-inhibition activity contribute to the protection of mice against lethal infection.. J Gen Virol.

[31] Gocník M, Fislová T, Mucha V, Sládková T, Russ G (2008). Antibodies induced by the HA2 glycopolypeptide of influenza virus haemagglutinin improve recovery from influenza A virus infection.. J Gen Virol.

[32] Sui J, Hwang WC, Perez S, Wei G, Aird D (2009). Structure and functional bases for broad-spectrum neutralization of avian and human influenza A viruses.. Nat Struct Mol Biol.

[33] Chen MI, Barr IG, Koh GCH, Lee VJ, Lee CPS (2010). Serological Response in RT-PCR Confirmed H1N1-2009 Influenza A by Hemagglutination Inhibition and Virus Neutralization Assays: An Observational Study.. PLoS One.

[34] Ross T, Zimmer S, Burke D, Crevar C, Carter D (2010). Seroprevalence following the second wave of pandemic 2009 H1N1 influenza.. PLoS Curr.

